# An intrinsic dynamics capture network for long-term airspace traffic prediction

**DOI:** 10.1371/journal.pone.0338949

**Published:** 2026-01-05

**Authors:** Bo Liu, Weizhen Tang, Zhousheng Huang

**Affiliations:** Civil Aviation Flight University of China, Guanghan, China; National University of Defense Technology, CHINA

## Abstract

With the rapid growth in global air traffic volume, accurate airspace traffic prediction has become critical for enhancing aviation safety and promoting sustainable airspace management. However, most existing approaches have demonstrated success primarily in short-term forecasting, whereas long-term traffic prediction remains challenging. This difficulty arises because, as the temporal granularity grows, the predictive model’s ability to learn traffic dynamics declines, and positional information is more easily lost over long sequences, resulting in diminished forecasting accuracy. To address these challenges, this paper proposes a novel intrinsic dynamics capture architecture, termed IDCformer, which capitalizes on the intrinsic characteristics of airspace flow to achieve long-term sequence prediction. IDCformer comprises three core modules: a Trend and Seasonal Extraction module (TSE), enhanced feature representation and position-aware Patch Time Series Transformer (PatchTST), and a Local Self-Attention module (LAT). Specifically, the TSE module preprocesses the input data to stabilize the data and extract long-term dynamics; second, the position-aware PatchTST alleviates the issue of temporal order loss in long sequences by integrating convolutional positional signals; finally, the LAT provides hierarchical refined processing to capture local fluctuations, thereby improving the accuracy of long-term forecasting. Experimental results based on real-world air traffic data indicate that our method surpasses other state-of-the-art models in predictive performance. Furthermore, this paper investigates the capacity of IDCformer to incorporate external information; the findings demonstrate that when external data are introduced as additional input features, IDCformer’s long-term prediction performance is further enhanced, illustrating its potential for effectively leveraging multisource information.

## Introduction

The air transport industry serves as a crucial hub connecting global regions, playing an irreplaceable role in promoting economic growth and enhancing cultural exchanges. However, the rapid increase in air traffic has posed unprecedented challenges to airspace traffic management [1–3]. Effective airspace traffic prediction is essential not only for ensuring aviation safety and optimizing resource allocation but also for enhancing the overall efficiency of the aviation system [4,5].

Long-term airspace traffic forecasts enable airlines to anticipate future traffic trends, facilitating more rational route planning [6]. While short-term forecasts are vital for real-time scheduling and emergency responses, they have a limited prediction range and struggle to address fluctuations and trends over extended periods. In contrast, long-term forecasts, covering time scales of several days [7,8], can better capture the impacts of weather changes, flight schedule adjustments, and holiday effects on airspace traffic. This approach aids airlines in optimizing flight scheduling and resource allocation and provides air traffic control (ATC) with more time for airspace planning and adjustments. Additionally, long-term forecasts assist in developing more reasonable air density control strategies, reducing delays and congestion, and improving the operational efficiency of the overall aviation system [9,10]. Therefore, developing accurate long-term airspace traffic prediction models is significant for enhancing the flexibility and responsiveness of aviation operations.

Currently, airspace traffic prediction primarily relies on historical data analysis, statistical models, and recent machine learning methods [11–13]. These approaches have achieved some success in short-term prediction but face limitations in long-term forecasting [14,15]. Firstly, traditional statistical models often assume linear data properties, making it difficult to capture complex nonlinear dynamic changes. Secondly, although machine learning methods excel in handling large-scale data and complex pattern recognition [16], their generalization ability in long-term trend and seasonal forecasting needs further improvement. Moreover, airspace traffic is influenced by various factors, including weather conditions and aviation policies, whose complex interactions increase the difficulty of long-term prediction [17,18].

In recent years, deep learning technology has emerged as a novel prediction tool in airspace traffic prediction research, demonstrating potential in handling complex data and nonlinear problems [19–21]. Unlike traditional statistical models, deep learning can automatically extract and learn complex relationships hidden within large-scale data through multi layer network structures and powerful parameter learning capabilities [22,23]. Several studies have recognized that airspace traffic is affected by multiple factors, such as weather variations, flight altitude, aviation policies, and holiday effects [24–26]. For example, Liu et al. [27] combined weather data to analyze the effects of different meteorological conditions, such as snowstorms and high winds, on airspace traffic flow, proposing a multifactor fusion prediction model. Han et al. [28] improved the prediction model by incorporating aviation policy and holiday data, demonstrating that these external factors significantly impact traffic flow and substantially improve prediction accuracy when introduced into the model. Additionally, Spatharis et al. [29] proposed an augmented learning-based traffic prediction model considering not only airspace traffic flow but also flight altitude and policy differences among airlines.

Despite the significant progress achieved by these advanced technologies, their performance in long-term forecasting still faces fundamental structural limitations. The primary challenges in long-term prediction are not the external factors themselves—which often act as valuable auxiliary variables—but rather the model’s intrinsic ability to capture long-term dependencies and mitigate error accumulation. For example, architectures reliant on recurrent or attention mechanisms, while powerful, often face inherent difficulties in preserving precise temporal context and avoiding information decay over the extended horizons required for long-term forecasting. While incorporating external factors can enhance performance, it cannot compensate for a core architecture that is ill-suited for these long-horizon tasks. Consequently, a critical challenge in this domain is to design a model architecture that is inherently robust to these long-term challenges, capable of maintaining predictive accuracy by effectively modeling the intrinsic dynamics of the traffic data itself.

To overcome the limitations of existing methodologies, this paper introduces a deep learning model specifically designed to analyze trends, fluctuations, and seasonality in airspace traffic dynamics. Specifically, a linear layer is utilized to capture the data’s linear relationships, while the data concurrently flows into a Trend and Seasonality Extraction (TSE) layer. This layer, comprising two convolutional networks, is tailored to extract nonlinear relationships, trends, and seasonal features from airspace traffic data. Leveraging the characteristics of convolutional operations and patching techniques, the processed features are divided into multiple patches embedded with implicit positional information. These patches are subsequently fed into a Transformer [30] encoder to enable the learning and capture of global patterns. Finally, a localized self-attention mechanism is applied to the globally learned features, enabling the model to capture short-term local fluctuations effectively and achieve comprehensive perception of airspace traffic data.

Overall, our study makes the following three main contributions:

1. To improve the prediction accuracy of long-term airspace traffic, we designed a network architecture that captures the internal dynamics of traffic from three synergistic aspects. First, a Trend and Seasonal Extraction (TSE) module pre-processes the data to stabilize and extract long-range dynamics, tackling the issue of declining long-term accuracy. Second, an enhanced position-aware PatchTST [31] mitigates the loss of temporal order over long sequences by integrating robust convolutional positional signals. Finally, a Local Self-Attention (LAT) module provides a hierarchical refinement, capturing short-term local fluctuations to improve the precision of the global forecast. This process of pre-extraction, positionally-aware encoding, and local refinement ensures both stability and accuracy in long-term predictions.
2. To investigate the model’s capacity to integrate external information, we systematically analysed exogenous variables, including flight altitude, heading angle, and latitude/longitude. By incorporating these factors as input features, we quantified the model’s ability to learn from external information. Interestingly, the experimental results indicate that the combined utilisation of multiple external variables does not always outperform the use of a single variable. This finding suggests potential redundancy or interference among the external variables.
3. Experiments on real-world air traffic datasets demonstrate that our proposed method outperforms state-of-the-art machine learning and deep learning methods.

## Literature review

Airspace traffic forecasting is crucial in aviation management, aiming to optimize air traffic resource allocation, reduce delays [32], and enhance operational efficiency [33] by predicting future traffic changes. The rapid growth of global air traffic has increased airspace complexity, making accurate traffic prediction increasingly important. Over the past decades, researchers have developed various methods to address this problem, ranging from traditional statistical models to modern machine learning and deep learning techniques. Each method has unique advantages and limitations, especially when facing the complex and dynamic airspace environment. Achieving traffic predictions that meet expected criteria has become a significant research challenge. To contextualize the specific challenges addressed in this paper, this review is organized as follows: we first touch upon the broader field of land-based traffic prediction, then survey methods for short-term air traffic forecasting, and finally focus on the advancements and remaining gaps in long-term air traffic forecasting.

### Land-based traffic flow prediction

Traffic flow prediction is a mature and extensively studied field, particularly in the context of land-based transportation systems such as urban road networks. Research in this area has progressed from traditional statistical models to machine learning and, more recently, deep learning approaches designed to handle complex spatio-temporal dependencies. State-of-the-art methods for ground traffic often employ Graph Neural Networks (GNNs) [34] to explicitly model the fixed topology of road networks, effectively capturing the intricate spatial relationships between interconnected roads and intersections. This is necessary because ground traffic is characterized by high stochasticity and complex, grid-like spatial dependencies influenced by numerous factors like traffic signals, accidents, and local events. In ground transportation safety research, two studies on U.S. North Carolina data focus on vulnerable road users (VRUs) [35,36]: one develops a priority-based framework using 2014–2019 data to identify high-risk locations, noting changing impacts of factors like traffic control (declining influence) and daylight (growing influence) on crash severity; the other uses nine-year data and seasonal random parameter models to find seasonal variations in pedestrian crash factors (e.g., hit-and-run in spring, alcohol impairment in summer) and higher crashes in darker seasons. Furthermore, recent studies have demonstrated the extensive potential of advanced deep learning and stochastic optimization frameworks in addressing complex challenges within intelligent transportation systems, ranging from infrastructure planning and anomaly detection to data synthesis under scarcity [37–41].

In contrast, airspace traffic is more highly regulated, follows more structured long-range trends, and its network topology is more fluid and less rigidly defined than a physical road graph [42,43]. These fundamental differences necessitate the development of specialized models tailored to the unique dynamics of the aviation domain, rather than simply adapting land-based models. This review will therefore focus on methods specifically applied to or suitable for air traffic forecasting.

### Short-term air traffic flow prediction

Short-term forecasting is essential for tactical air traffic management, focusing on real-time adjustments and safety assurance. In the early stages, traditional statistical models were widely used for this purpose. For example, Nieto et al. [44] employed Autoregressive Integrated Moving Average (ARIMA) models to effectively capture short-term traffic fluctuations in low-complexity environments. Similarly, the dynamic updating capability of Kalman filtering [45] has been applied in air traffic control to adjust predictions in real time by incrementally updating flight data, providing timely and accurate forecasts in response to immediate changes [46].

More recently, deep learning methods have been applied to short-term tasks. Lin [47] proposed a model based on ConvLSTM modules to accurately predict flow distributions at varying altitudes, while Jardines [48] developed a Convolutional Neural Network (CNN)-based model to predict thunderstorms, enhancing tactical air traffic planning. To handle the complex network topology of the airspace system in real time, some studies have used Graph Convolutional Networks (GCNs) to effectively model the intricate relationships between nodes in large-scale airspace systems [49]. In parallel, other advanced Transformer-based architectures, such as the Temporal Fusion Transformer (TFT), have been successfully applied to predict numerical airport arrival delays at a high temporal resolution, demonstrating strong performance with multi-factor inputs [50]. Valuable methodological insights can also be drawn from the adjacent domain of urban rail transit (URT), where researchers have developed sophisticated models to handle sharp, non-routine traffic fluctuations during events, holidays, or public health emergencies. These works demonstrate the efficacy of advanced deep learning architectures for such scenarios. For instance, Transformer-based models have been designed to explicitly separate and predict the “extra” passenger flow generated by large-scale events. Furthermore, Graph Neural Networks (GNNs) have been extended to not only capture spatial dependencies but also to integrate multi-frequency temporal patterns (e.g., real-time, daily, and weekly) and even incorporate physics-guided loss functions to enhance model interpretability. Other approaches have utilized multi-task learning frameworks to jointly predict related variables like station inflow and outflow, capturing their complex interactions [51–54]. While effective for immediate operational needs, these methods are primarily designed for tactical decision-making and often struggle to capture the broader patterns required for long-range strategic planning.

### Long-term air traffic flow prediction

Long-term forecasting, which covers time scales of several days, is critical for strategic planning, including optimizing airline schedules and airspace resource allocation. This task requires models that can effectively capture underlying trends and seasonality. Traditional methods like the Holt-Winters technique have been used to forecast aggregated passenger data by decomposing the time series into trend and seasonal components [55].

To handle the non-linearities inherent in long-term data, machine learning and deep learning models have become the primary focus. Ensemble learning methods such as Random Forests have been used to analyze features like flight origins and destinations to predict air traffic flows and flight levels [12]. Deep learning models, which excel at handling complex long-term dependencies, have also been a major area of research [56–58]. For instance, Gui et al. [59] applied Long Short-Term Memory (LSTM) networks to airway traffic prediction, demonstrating strong performance, particularly when abnormal factors are considered.

A more recent and transformative trend is the application of Large Language Models (LLMs) to spatio-temporal forecasting. This emerging field aims to create spatio-temporal foundational models with strong generalization capabilities. For example, UrbanGPT [60] has been proposed to integrate spatio-temporal dependency encoders with the instruction-tuning paradigm, enabling LLMs to make accurate predictions even in data-scarce or zero-shot scenarios. Alongside the development of new models, efforts are also underway to create comprehensive benchmarks, such as STBench [61], to systematically evaluate the spatio-temporal knowledge, reasoning, and application capabilities of existing LLMs.

To further improve long-term accuracy, researchers have increasingly incorporated external variables, such as weather conditions and holidays, into deep learning models [62]. However, while these advanced models have shown progress, they face significant challenges. As the number of input features grows, the computational complexity and resource requirements escalate rapidly. Furthermore, for high-dimensional inputs and multi-layer neural networks, the total number of parameters can expand swiftly, increasing model complexity without guaranteeing better performance. There is an urgent need for a model that can efficiently and effectively learn the fundamental nonlinear variations, trends, and seasonal patterns from the airspace traffic data itself, forming the primary motivation for this work.

## Problem formulation

The historical traffic data for a single, predefined airspace can be represented as a two-dimensional tensor $X\in R^{T\times d}$. Each element $x_{T,d}$ in this tensor corresponds to the value of the d-th feature at the t-th time step. The feature dimension, d, includes variables representing the airspace traffic flow. This historical data is fused with other relevant feature sequences (e.g., flight traffic state such as flight altitude and heading) via concatenation at each time step to form an integrated feature matrix, $\overset{\sim }{X}$. If the additional feature sequence has a dimension of $d_{e}$, the resulting feature dimension of the integrated matrix becomes ${d+d}_{e}$.

Our goal is to use the historical integrated feature sequence $\overset{\sim }{X}$ to predict the airspace traffic over a future prediction horizon of P time steps. This sequence of future predictions is denoted as $\overset{\wedge }{y}=\{{\overset{\wedge }{y}}_{t+\text{1}},{\overset{\wedge }{y}}_{t+\text{2}},...,{\overset{\wedge }{y}}_{t+P}\}$. Each value ${\overset{\wedge }{y}}_{t+k}$ in this sequence represents the predicted number of flights in a particular airspace or altitude stratum at the future time step t+k.

Essentially, airspace traffic prediction can be viewed as finding a suitable mapping function F between historical and future data that enables it to predict future traffic based on a sequence of historical composite features $\overset{\sim }{X}$: $\overset{\wedge }{y}=F(\overset{\sim }{X};\theta )$, θ are model parameters that need to be learnt from historical airspace data; F is a predictive model designed to capture the complex spatio-temporal dependencies of airspace traffic.

## Model architecture

### Overall structure

**Fig 1:**
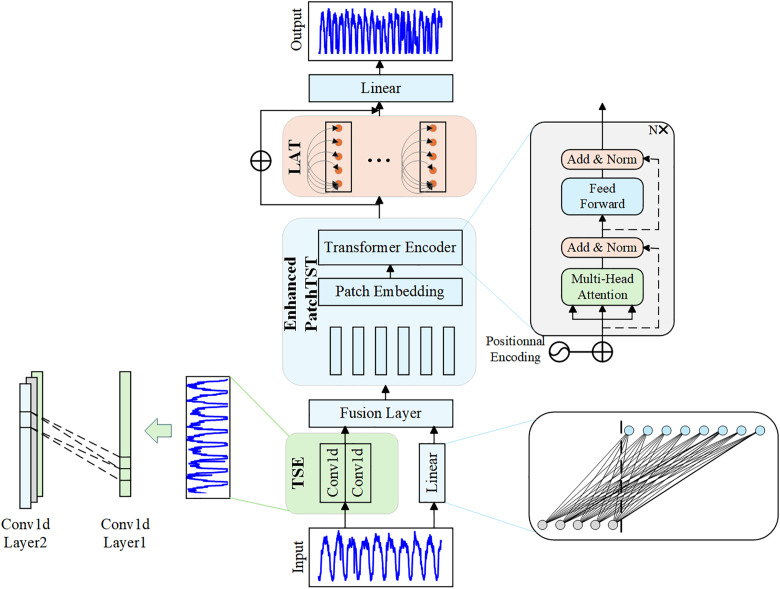
The overall architecture of the proposed IDCformer model.

This paper introduces a deep learning model, IDCformer, specifically designed for long-term airspace traffic prediction. As depicted in Fig 1, the model builds upon the transformer architecture, incorporating multiple feature extraction techniques. The extracted features are divided into patches enriched with positional information, enabling stronger feature representation and positional awareness capabilities. IDCformer is composed of several modules, including the Trend and Seasonality Extraction (TSE) module, the PatchTST module with enhanced feature representation and positional awareness, and the Local Attention Transformer (LAT) module.

IDCformer aims to enhance the modeling capability for complex air traffic flow data through multi-module feature extraction and patch-based processing. Specifically, the processing workflow for the input sequence is as follows: First, the input sequence undergoes parallel processing, where linear transformations are applied, and the Trend and Seasonality Extraction (TSE) module captures trend and seasonal features. Next, the processed feature representations are segmented into equal-length patches using patching techniques and mapped to a high-dimensional space. Subsequently, a Transformer encoder is employed to extract global dependencies. Building on this, a local attention mechanism is utilized to strengthen local feature representations. Finally, the predicted air traffic flow is obtained through a linear layer.

### Feature extraction module

The feature extraction module learns the linear relationship of the data through two sub-modules, Linear and TSE, on the raw input series respectively, and captures the characteristics of the flow changes in terms of global trends and seasonality.

The Linear module extracts the implicit information directly from the sequence level through a two-layer linear mapping, and uses the neural net layer to process the linear relationships in the original sequence. For linear transformation, we define the input sequence $X\in R^{T\times d}$ is linearly transformed as:


$$Y_{linear}=XW+b$$ (1)

where $W\in R^{d\times d^{\prime }}$ is the weight matrix of the linear mapping and $b\in d^{\;\;\prime }$ is the bias vector.

**Fig 2:**
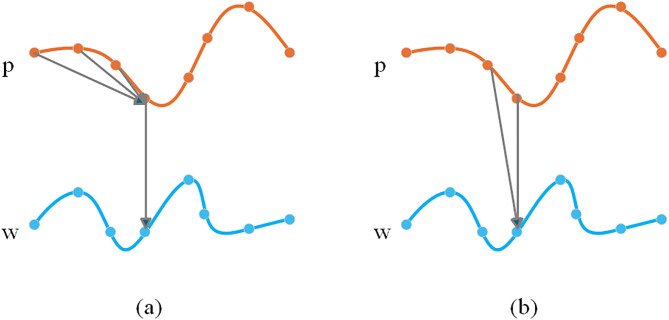
Comparison between CNN-based Methods and RNN-based Methods. (a) CNN-based Methods: Node w acquires and fuses information from its neighboring node p within a short time window at the current and preceding time steps to efficiently capture trends and seasonality. (b) RNN-based Methods: At the current time step, node w receives raw information from its neighboring node p and integrates latent representations from the previous time step to capture temporal features.

In time series analysis, trend and seasonality features typically manifest as correlations between neighbouring time points. Convolutional operations effectively learn smooth trends and seasonal patterns in time series due to their local connectivity and parameter-sharing properties. Fig 2 compares Recurrent Neural Network (RNN)-based methods and Convolutional Neural Network (CNN)-based methods, with Fig 2(a) clearly demonstrating the superiority of convolution in capturing trends and seasonal features.

As shown in Fig 2(a), the CNN-based method processes information in parallel. A convolutional filter aggregates weighted information from multiple neighbouring points within a local window simultaneously. This parallel, windowed view allows the model to directly perceive the local shape and structure of the time series, making it highly effective at identifying local slopes (trends) and periodic patterns (seasonality). In contrast, Fig 2(b) depicts the RNN-based method, which processes information sequentially. At each time step, it considers only the current input and its own past hidden state. While excellent for capturing long-term temporal dependencies, this point-by-point processing is less direct for identifying local shapes, as an RNN must infer a pattern by remembering a sequence of individual points, whereas a CNN recognizes the pattern of that sequence in a single operation. Therefore, for the specific task of extracting trend and seasonality, the convolutional approach is inherently more direct and efficient.

For sequential data, one-dimensional convolution simplifies computation by avoiding the need to process additional dimensional information, unlike two-dimensional convolution. Furthermore, as the convolution kernel slides along the temporal axis, the extracted features at each time step vary according to the local context at that specific position, inherently making the convolution operation position aware. Consequently, our TSE module employs one-dimensional convolution to capture long-term trends and seasonal components in traffic data. This process is represented as:


$$Y_{TSE}=Conv\text{1}D(X;K,P)+P_{I}(X)$$ (2)

Where, K is the convolution kernel, P is the padding parameter and $P_{I}(X)$ is the implicit position information carried by the 1D convolution operation. Specifically, to effectively capture both long-term trends and shorter-term seasonality, the TSE module consists of two parallel 1D convolutional layers, organized into a two-branch structure. The first branch, designed for trend extraction, employs a 1D convolutional layer with a larger kernel size (48) to smooth the sequence and capture low-frequency signals. The second branch focuses on seasonality, using a 1D convolutional layer with a smaller kernel size (16) to identify more local, periodic patterns. Following each convolutional layer, a GELU (Gaussian Error Linear Unit) activation function is applied to introduce non-linearity. The outputs from these two branches are then fused through element-wise addition, allowing the model to learn a combined representation of both trend and seasonal dynamics.

The final linearly transformed output with the combination of trend and seasonal components is:


$$Y_{feature}=Y_{linear}+Y_{TSE}$$ (3)

Through the collaboration of these two modules, the model extracts richer features of flow changes, thereby effectively enhancing its ability to learn from data.

### Enhanced feature representation and position-aware PatchTST

PatchTST is a Transformer-based model for long-term time series forecasting, introduced in recent years. By segmenting the original sequence into patches and leveraging the Transformer’s global attention mechanism, PatchTST effectively models long-range dependencies across patches, enabling accurate long-term time series predictions.

In this paper, we enhance the long-term prediction capability of PatchTST by strengthening the feature representation of Patch and injecting position information into it. Specifically, the core idea of the original PatchTST is to divide the original time series X into a number of non-overlapping patches $\left\{X^{\text{1}},X^{\text{2}},...,X^{N}\right\}$, then perform linear mapping (i.e., Patch Embedding) on each $X^{N}$, and finally embed all the patches into the input Transformer encoder for global modelling. However, directly dividing the raw sequence into a number of patches would result in redundant information or noisy features, making it difficult for the model to effectively discriminate the correct feature representation. The enhanced PatchTST in this paper is to divide the high-level feature representation $Y_{feature}\in R^{T\times d^{\prime }}$ in Eq. (3) into $N=\frac{T}{P}$ patches with implicit position information:


$$Y_{patch}=\{Y_{\text{1}},Y_{\text{2}},...,Y_{N}\}$$ (4)

Before doing patch division, the TSE module in Eq. (2) captures a more advanced and smoother representation of the temporal features, which allows the divided $Y_{patch}$ to have clearer information in the time dimension and smoother articulation between patches. Where each patch $Y_{i}\in R^{P\times d^{\prime }}$ can be represented as:


$$Y_{i}=Y_{feature}[(i-\text{1})P+\text{1}:iP,:]$$ (5)

Each patch is then mapped to a higher dimensional space via an embedding matrix:


$$z_{i}=Y_{i}W_{embed}+b_{embed}$$ (6)

Where, $W_{embed}$ denotes the embedding matrix and $b_{embed}$ is the bias term, after the above processing, the embedded feature sequence can be obtained:


$$Z=[z_{\text{1}},z_{\text{2}},...,z_{N}]\in R^{N\times P\times d_{e}}$$ (7)

This process involves segmenting the sequences, organizing them into multiple patches, and embedding them into sequences that the Transformer can learn from and subsequently pass to the Transformer encoder. The patch size is a critical hyperparameter that determines the granularity of the input to the Transformer. A size that is too large can average out important short-term patterns, while a size that is too small increases computational cost and may lose local contextual information. Therefore, in this paper, we adopt a patch size of 16, which is commonly used in relevant studies.

After embedding the patch, the original PatchTST applies learnable positional encoding to capture the temporal positions within the time series. Meanwhile, the convolutional property of the TSE module, as described in Eq. (2), inherently provides relative positional information for neighbouring moments. This process is equivalent to embedding linearly refined local positional signals. The integration of these positional encodings further enhances PatchTST’s ability to sense position and temporal patterns in traffic data, as represented by:


$$P_{final}=PE+P_{I}(X)$$ (8)

where $P_{final}$ denotes the final position information and PE denotes the position information of the learnable position code. This learnable positional encoding is implemented as an embedding layer, where each position in the sequence of patches is assigned a unique vector that is optimized during training. This positional information is then combined with the patch embedding via element-wise addition. For this operation to be possible, the embedding size for both the patch and the positional encoding must be identical, which is set to 128 in our model. capacity:e of embedding size affects the model’s capacity; a size too small may lead to underfitting, while one too large increase the risk of overfitting and computational cost. Therefore, to avoid model overfitting and save computational costs, the embedding dimension is set to 128.

The global features are then extracted using a Transformer encoder, which models the long-range dependencies in traffic data through a multi-head self-attention mechanism. Each patch is processed through multiple layers of Transformer encoders. By calculating the correlations between sequence elements, the self-attention mechanism generates a weight matrix that is used to compute a weighted sum of the value vectors, producing a new representation while emphasizing distinct subspace features. Furthermore, the Transformer encoder incorporates a feed-forward neural network, enabling independent linear transformations of the representations at each position, thereby enhancing the model’s representational capacity.

### Local self-attention

Although a global self-attention mechanism is applied to patches to capture long-term dependencies and semantic relationships, the local associations between patches are insufficiently addressed. This limitation arises because the Transformer encoder primarily applies attention between patches, hindering the model’s ability to detect local fluctuations effectively. To overcome this, we design a local attention mechanism that employs window segmentation to enable finer-grained interactions at a local scale, building upon features that already contain global contextual information.

While our LAT module utilizes a self-attention mechanism, it is fundamentally different from the global attention in the main Transformer encoder in its scope, architectural role, and the data it processes. Unlike the main encoder’s attention, which operates globally across all patches to learn long-range dependencies, the LAT module is a subsequent, refining step that operates locally within fixed, non-overlapping windows to capture short-term fluctuations. Critically, it takes the globally aware feature representations from the main encoder as its input, rather than the initial embeddings. This hierarchical approach, which first models the global trend and then focuses on local details, enables the model to achieve long-term stability without compromising local accuracy.

Following the modelling of long-range dependencies in PatchTST, short-term fluctuations in airspace traffic data are captured through the local self-attention mechanism. With a daily data step of 96 and a patch size of 16, the window size is set to 6, corresponding to the time step of the preceding day. Within each window, the multi-head self-attention process is applied. The specific self-attention computation is as follows:


$$Q_{i},K_{i},V_{i}=W_{q}W_{i},W_{k}W_{i},W_{v}W_{i}$$ (9)

Where $Q_{i}$, $K_{i}$, $V_{i}$ are Query, Key and Value matrices respectively. They are obtained by linear transformation. $W_{i}$ are the feature representations of the input window, and $W_{q}$, $W_{k}$, $W_{v}$ are the weight matrices of Query, Key, and Value, respectively. The self-attention calculation formula is as follows:


$$Attention(Q_{i},K_{i},V_{i})=softmax(\frac{Q_{i}K_{i}^{T}}{\sqrt{d_{k}}})V_{i}$$ (10)

where $d_{k}$ is the scaling factor, usually equal to the dimension of the key vector, used to stabilize the gradient. The attention weight matrix is generated by computing the dot product of the query and the keys, which is then normalized by SoftMax. Finally, this attention matrix is used to generate a new feature representation by adding a vector of weights.

Local self-attention is primarily used for short-term temporal dependencies, thereby enhancing the model’s capacity to capture local patterns. Following the application of local self-attention, the model integrates the outputs with the global features extracted by the Transformer encoder via residual connections.


$$Z_{final}=Z+Attention(Z)$$ (11)

Where Z is the global feature representation, the residual linkage effectively combines global and local information, enabling the model to have both an understanding of global dependencies and a keen capture of local variations.

Finally, the model performs an aggregation operation of the fused feature sequences using average pooling to obtain unified feature representation:


$$\begin{array}{c} f=\frac{\text{1}}{N}\sum {\mathrm{\backslash nolimits}}_{i=\text{1}}^{N}Z_{\left(i\right)}^{final} \end{array}$$ (12)

where $Z_{\left(i\right)}^{final}$ is the *i* element in the fused feature sequence. Finally the aggregated feature representation is mapped to the final prediction through a linear layer:


$$\overset{\wedge }{y}=fW_{out}+b_{out}$$ (13)

Where $W_{out}$ is the weight matrix of the output layer and $b_{out}$ is the bias term.

Through the above process, the model in this paper is able to make full use of the self-contained features of the input sequences themselves to capture the complex temporal dependencies and achieve multi-step prediction of airspace traffic.

## Experiment

### Dataset and experimental settings

In this section, we use real-world airspace traffic data to evaluate the performance of the proposed model. The dataset comprises real-time ADS-B data collected at 15-minute intervals from 1 June 2018–30 June 2018 within a 300 km radius centered on Frankfurt Airport. This includes flight data for all flights within this airspace. Specifically, the data encompass information such as flight ID, flight altitude, flight heading angle, and the latitude and longitude of each aircraft. ADS-B data acquisition and transmission can be affected by various factors, including signal occlusion and multipath effects, leading to data loss. Therefore, interpolation was employed to impute missing data during the preprocessing stage.

The dataset was divided into training, validation, and test sets in the ratio of 7:1.5:1.5. The TSE module consists of two parallel 1D convolutional layers, organized into a two-branch structure. There are three layers of Transformer encoders, and the number of attention heads matches the number of local attention heads in the final part of the model, which is 4. In the subsequent LAT module, local attention is applied with a window size of 6 using non-overlapping windows. The resulting local features are then integrated with the global features from the main encoder via a residual connection, as described in the “Local self-attention” section. The batch size was set to 64 to ensure robust model performance on the dataset.

**Fig 3:**
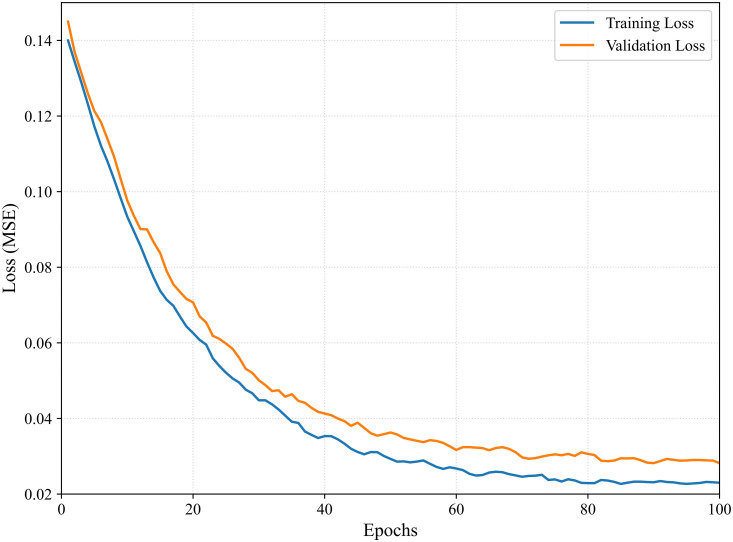
Training and validation loss curves over 100 epochs.

To ensure robust convergence and mitigate overfitting, we implemented a rigorous training strategy. The model was trained using the Adam optimizer with an initial learning rate of 1e-3. We employed a Cosine Annealing learning rate scheduler, which gradually decreases the learning rate to a minimum of 1e-6 over the course of training, facilitating smoother convergence in later epochs. The batch size was set to 64. The maximum number of training epochs was set to 100. To further prevent overfitting, we adopted an early stopping strategy with a patience of 10 epochs; training was automatically terminated if the validation loss did not improve for 10 consecutive epochs. Fig 3 presents the training and validation loss curves. As shown, both curves descend rapidly from an initial value of approximately 0.14 and converge to a low error range after 80 epochs. The consistent alignment between training and validation loss demonstrates the stability of the training process and the effectiveness of the regularization strategy. All experiments were conducted on a desktop computer equipped with a 13th Gen Intel® Core™ i7-13700H processor, 16 GB of RAM, and an NVIDIA GeForce RTX 4060 Desktop GPU.

### Evaluation metrics

We divide the whole airspace traffic data into three common airspaces according to altitude: high altitude airspace (above 6000m), medium altitude airspace (3000m ~ 6000m) and low altitude airspace (below 3000m). Consequently, the objective was to predict flight traffic not only across the entire airspace but also within these three specific airspaces to provide controllers with more detailed decision-making guidance. Additionally, long-term traffic prediction in the airspace involves multiple time steps; therefore, Root Mean Square Error (RMSE), Mean Absolute Error (MAE), and Mean Absolute Percentage Error (MAPE) were employed to evaluate the model’s performance.


$$\begin{array}{c} \text{RMSE}=\sqrt{\frac{\text{1}}{n}\sum {\mathrm{\backslash nolimits}}_{i=\text{1}}^{n}{\left(y_{i}-{\hat{y}}_{i}\right)}^{\text{2}}} \end{array}$$ (14)

$$\begin{array}{c} \text{MAE}\;=\frac{\text{1}}{n}\sum {\mathrm{\backslash nolimits}}_{i=\text{1}}^{n}{\left(y_{i}-{\hat{y}}_{i}\right)}^{\text{2}} \end{array}$$ (15)

$$\begin{array}{c} \text{MAPE}=\frac{\text{1}}{n}\sum {\mathrm{\backslash nolimits}}_{i=\text{1}}^{n}\left|\frac{y_{i}-{\hat{y}}_{i}}{y_{i}}\right|\times \text{100}\% \end{array}$$ (16)

Where: n denotes the sequence length of the airspace traffic data, $y_{i}$ is the ADS-B data value, and ${\hat{y}}_{i}$ is the airspace traffic prediction value. To interpret these metrics, it is crucial to understand that the prediction target, $y_{i}$, is a single scalar value: the total number of aircraft in a defined airspace. Therefore, the metrics directly quantify the error in this aircraft count prediction.

The practical significance of this is best explained using MAE, which represents the average absolute difference between the predicted and actual aircraft counts. For the 24-hour prediction (Table 1 in the subsequent “Model Comparison” section), the baseline HA model has an MAE of 106.98, meaning its prediction is, on average, wrong by about 107 aircraft. In stark contrast, our IDCformer model achieves an MAE of 45.19, meaning its average error is only about 45 aircraft. This improvement from an average error of 107 aircraft to 45 is practically significant. It elevates the prediction from a rough estimate to a reliable decision-support tool, enabling air traffic managers to proactively optimize sector configurations and staffing with much greater confidence, thereby enhancing operational safety and efficiency.

**Table 1:** Performance of each model in entire altitude airspace, high-altitude airspace, mid-altitude airspace, and low-altitude airspace. Where dark colored numbers are the best performance and underlined numbers are the second best.

Data	Model	24h			48h			72h			96h		
		RMSE	MAE	MAPE	RMSE	MAE	MAPE	RMSE	MAE	MAPE	RMSE	MAE	MAPE
Entire	HA	137.89	106.98	11.85	145.74	110.07	10.75	155.05	137.82	11.86	161.46	140.18	14.37
	SVR	73.74	57.22	7.94	115.84	78.54	12.23	118.54	79.32	12.97	134.16	86.09	15.24
	LSTM	70.93	58.35	7.86	91.16	70.63	9.01	94.99	70.45	9.59	98.57	73.37	9.63
	Autoformer	65.12	51.98	6.37	83.77	65.78	8.4	85.35	67.87	9.37	89.82	71.87	9.39
	PatchTST	56.83	45.69	5.99	75.06	61.77	7.61	75.33	61.39	8.07	77.79	61.93	7.36
	iTransformer	55.62	46.37	5.81	78.13	62.22	8.34	79.28	64.91	8.28	81.22	67.61	7.94
	ModernTCN	55.97	45.33	5.94	**70.29**	**54.72**	**7.44**	77.59	64.84	8.2	80.07	63.97	7.44
	AMD	57.01	45.92	6.05	75.34	62.03	7.68	76.85	62.73	8.35	79.26	63.15	7.68
	IDCformer	**54.29**	**45.19**	**5.58**	73.81	56.91	7.57	**73.12**	**61.03**	**7.89**	**76.85**	**59.26**	**7.16**
High-altitude	HA	134.04	99.51	12.2	145.65	110.18	13.66	148.99	117.64	22.05	163.23	124.43	15.17
	SVR	61.37	47.72	7.57	90.14	68.58	15.38	101.83	72.15	17.43	105.85	74.79	10.78
	LSTM	62.56	46.12	8.36	75.15	52.97	13.65	85.76	58.24	14.57	85.41	61.03	13.96
	Autoformer	54.41	41.18	6.29	60.62	46.33	7.18	73.04	53.7	7.93	75.31	59.47	7.95
	PatchTST	49.57	40.21	5.92	58.43	46.88	6.6	65.83	**45.67**	7.25	73.25	57.74	8.07
	iTransformer	46.51	38.09	5.55	56.03	43.56	6.34	69.52	50.56	7.72	75.11	60.06	8.24
	ModernTCN	49.08	39.18	5.95	55.92	43.09	6.04	**62.39**	47.16	7.01	66.83	54.81	7.93
	AMD	50.15	40.88	6.13	57.91	46.15	6.75	66.24	46.05	7.31	71.81	56.02	8.02
	IDCformer	**46.15**	**36.63**	**5.44**	**52.81**	**40.96**	**5.86**	63.13	46.74	**6.89**	**66.48**	**52.63**	**7.18**
Mid-Altitude	HA	34.62	26.33	39.48	33.31	25.47	29.55	33.15	24.96	27.04	40.02	30.78	31.74
	SVR	31.11	24.08	24.41	34.05	24.71	24.3	33.32	24.66	22.83	38.77	28.41	24.56
	LSTM	28.01	23.23	23.77	30.43	23.85	24.44	32.27	24.09	22.56	32.61	27.44	25.82
	Autoformer	24.39	20.13	22.38	26.59	21.84	26.53	26.11	21.8	24.05	27.3	22.87	23.39
	PatchTST	21.55	17.64	20.08	23.78	21.16	21.27	24.51	**19.57**	21.85	25.27	20.41	20.39
	iTransformer	21.88	18.94	20.18	23.75	20.88	22.09	24.94	21.54	21.88	25.78	21.61	21.45
	ModernTCN	23.58	17.26	20.91	25.63	21.51	23.52	24.65	22.44	**21.51**	26.63	21.83	22.19
	AMD	21.98	17.95	20.25	24.05	21.42	21.55	**24.28**	19.91	22.03	25.61	21.73	21.66
	IDCformer	**20.55**	**16.97**	**19.59**	**23.73**	**21.04**	**21.23**	24.86	21.32	21.64	**24.95**	**19.68**	**20.01**
Low-Altitude	HA	50.08	37.18	26.79	48.92	36.09	25.64	52.13	37.16	31.01	48.83	34.74	27.69
	SVR	30.54	23.16	21.46	44.71	28.9	30.92	42.99	28.51	27.33	49.71	33.18	29.18
	LSTM	25.04	20.93	17.33	29.58	20.85	16.89	30.94	23.26	23.64	33.4	25.6	23.96
	Autoformer	19.24	15.67	18.22	23.22	17.83	16.26	25.23	18.18	21.59	26.28	21.94	21.62
	PatchTST	16.12	12.94	12.18	19.87	13.86	13.32	22.97	17.88	19.85	23.61	18.99	18.31
	iTransformer	16.01	13.2	12.95	20.51	14.09	13.21	23.95	17.97	20.13	23.3	17.54	18.69
	ModernTCN	17.66	13.9	12.41	21.52	15.66	14.47	23.21	18.64	19.96	23.28	17.59	18.56
	AMD	16.35	13.11	12.34	20.01	14.05	13.58	23.15	17.85	19.74	23.89	19.01	18.49
	IDCformer	**15.69**	**12.55**	**12.07**	**19.83**	**13.64**	**13.18**	**22.15**	**17.75**	**19.67**	**23.13**	**17.28**	**18.06**

### Baseline methods

To comprehensively assess the model’s performance, five state-of-the-art baseline methods were selected for comparison, encompassing both machine learning and deep learning approaches:

(1) HA: Historical Average. It uses the average value of historical data to predict future values.
(2) SVR [63]: a support vector regression with linear kernel.
(3) LSTM [64]: Long Short-Term Memory. A specialized form of recurrent neural network, LSTM addresses the issues of gradient vanishing and explosion in traditional RNNs by introducing a gating mechanism, thereby effectively capturing long-term dependencies in sequences.
(4) Autoformer [65]: Based on the transformer architecture, it incorporates a series decomposition module and an auto-correlation mechanism.
(5) PatchTST [31]: Patch Time Series Transformer. A Transformer-based model that segments time series data into several patches, each containing a sequence of consecutive time steps and treated as a token.
(6) iTransformer [66]: An inverted Transformer model that performs inverted modelling of time series within an encoder-only framework, deeply exploring the correlations between variables through an attention mechanism.
(7) ModernTCN [67]: Modern Temporal Convolutional Network. An advancement of Temporal Convolutional Networks (TCN), ModernTCN enhances the receptive field of the convolutional kernel and adopts a lightweight design while maintaining performance.
(8) AMD [68]: An MLP-based framework that decomposes time series into multiple scales and adaptively synthesizes predictions. Its core is an Adaptive Multi-predictor Synthesis (AMS) block configured with several predictors.

These baseline models have undergone extensive research and practical validation, occupying a core position in the field of traffic flow prediction and demonstrating stable performance and strong adaptability in scenarios similar to air traffic. Incorporating them into comparative experiments allows coverage of a wide range of classical algorithmic approaches and advanced technical solutions. To ensure a fair comparison of air traffic flow prediction performance, the input sequence length of all baseline models is kept consistent with that of IDCformer.

### Model comparison

Table 1 presents the performance of the proposed method alongside other baseline methods across different test datasets, illustrating the variation in error for each model in predicting various time steps within different airspace traffic datasets. The analysis focuses on the following aspects:

Superiority of Deep Learning Models in long-term prediction: A comparative analysis of classical deep learning models, such as LSTM, and SVR on several datasets reveals that the prediction error of LSTM is only greater than that of SVR for relatively short time steps. As the prediction horizon increases, the error of LSTM becomes significantly smaller than that of SVR, while the errors of other state-of-the-art deep learning models are considerably lower than that of LSTM. This indicates that deep learning models are better suited for long-term predictions of flight traffic across different spatial domains. They effectively learn complex fluctuation patterns from large volumes of ADS-B data, primarily due to the deeper network structures and more sophisticated iterative mechanisms employed in deep learning models compared to SVR.

Accuracy of IDCformer in long-term forecasting: We compared the proposed model with state-of-the-art deep learning approaches, including ModernTCN, PatchTST, AMD and iTransformer. As shown in Table 1, the proposed model consistently outperforms the competing models across all time scales. Specifically, compared with the second-best model, PatchTST, IDCformer achieves overall reduction of 4.12% in RMSE, 4.20% in MAE, and 4.87% in MAPE. These results demonstrate that the proposed model is better suited for predicting long-term airspace flight traffic, owing to its superior ability to effectively capture the dynamics of airspace traffic conditions.

The stability and statistical significance of IDCformer: To rigorously evaluate our model’s ability to learn in a stable and reliable manner from the overall traffic flow, we conducted both 5-fold time-series cross-validation and hypothesis testing on the entire altitude dataset. The cross-validation results, presented in –, demonstrate that the proposed IDCformer not only consistently achieves the lowest average error across all metrics and prediction horizons but also exhibits small error bars. This indicates that its superior performance is stable and robust when evaluated on different partitions of the data. Furthermore, to formally verify that this outperformance is not due to random chance, hypothesis testing was conducted against the top three baseline models. As shown in Table 2, the p-values from the Diebold-Mariano test are consistently below the 0.05 significance level. This provides strong statistical evidence that the IDCformer’s performance represents a significant and reliable improvement over other advanced methods for long-term forecasting of the entire airspace’s traffic.

**Table 2:** Results of the Wilcoxon signed-rank test comparing IDCformer against advanced baseline models on the entire altitude airspace dataset. The values represent the p-values for each comparison. Bolded values indicate a statistically significant difference (p < 0.05).

Comparison Model	24h	48h	72h	96h
AMD	**0.011**	**0.014**	**0.014**	**0.012**
iTransformer	**0.035**	**0.017**	**0.028**	**0.032**
ModernTCN	**0.025**	0.061	**0.019**	**0.020**

**Fig 4:**
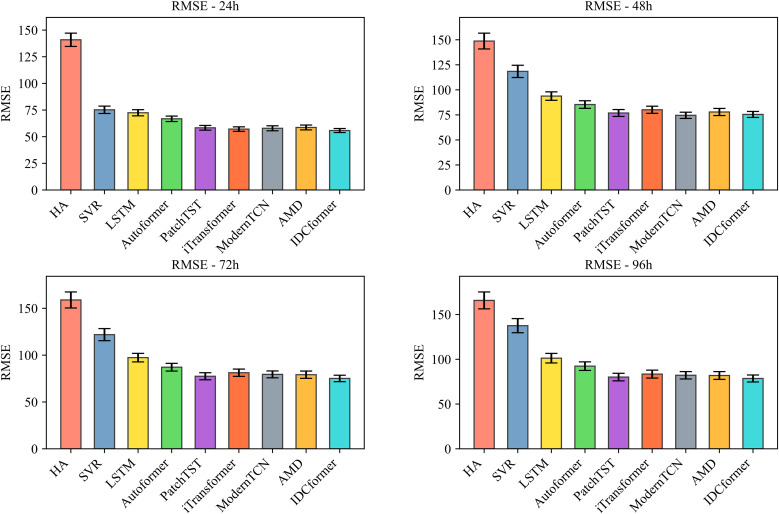
Comparison of RMSE for Various Models Under 5-Fold Time-Series Cross-Validation (where bar represents average errors and error bars denote standard deviations).

**Fig 5:**
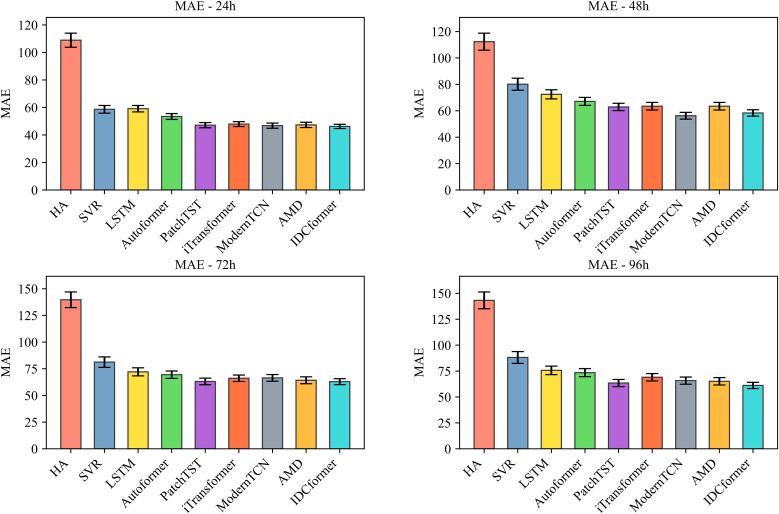
Comparison of MAE for Various Models Under 5-Fold Time-Series Cross-Validation (where bar represents average errors and error bars denote standard deviations).

**Fig 6:**
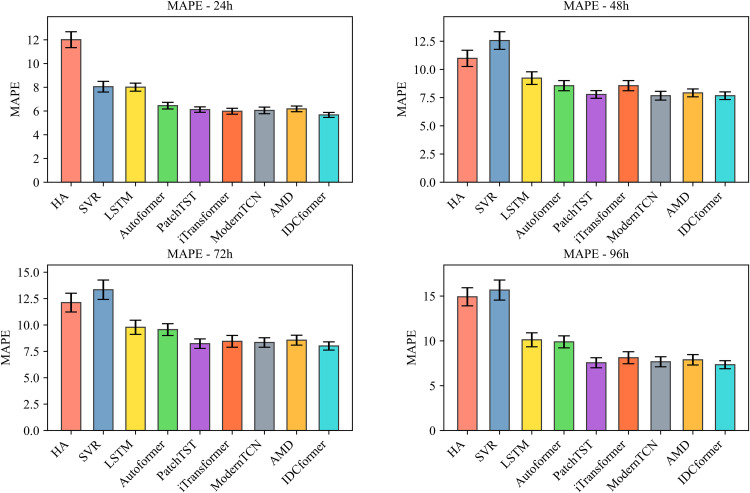
Comparison of MAPE for Various Models Under 5-Fold Time-Series Cross-Validation (where bar represents average errors and error bars denote standard deviations).

**Fig 7:**
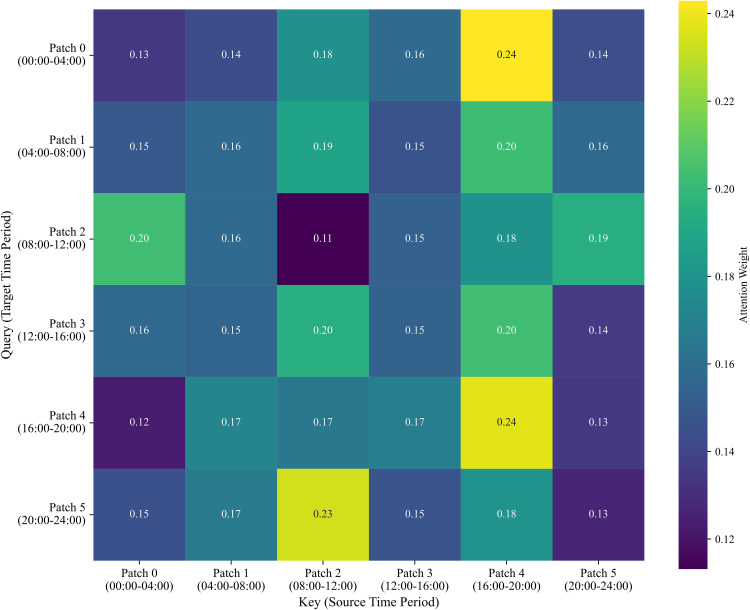
Integrated attention weight heatmap illustrates the intrinsic dynamics across different time periods of the day.

To comprehensively understand the interpretability of the proposed model, Fig 7 exhibits the integrated attention weight heatmap across different time periods of the day. This heatmap is derived by averaging the weights from the enhanced feature representation and position-aware PatchTST and the Local Attention module, providing a holistic view of the model’s focus. The vertical and horizontal axes denote the indices of query patches and key patches, respectively, where each patch corresponds to a 4-hour interval within the 96-step daily sequence (e.g., Patch 0 represents 00:00–04:00). Color brightness corresponds to attention weight magnitudes, with brighter colors indicating a higher degree of dependency and focus.

The visualization reveals that the model captures intrinsic dynamics by identifying critical periodic structures rather than distributing attention uniformly. Specifically, the model assigns the highest attention weights (reaching 0.24) to Patch 4 (16:00–20:00), identifying the evening peak as a deterministic anchor for the day’s traffic pattern. Furthermore, a significant structural “look-back” mechanism is observed: at the end of the sequence (Patch 5), the model heavily attends to Patch 2 (08:00–12:00) with a weight of 0.23. This demonstrates that IDCformer effectively utilizes the daily periodicity of airspace traffic, querying historical morning peak data to calibrate predictions for the night, thereby validating its ability to learn complex intrinsic dynamics beyond simple temporal proximity.

### Visual analytics

The MAPE effectively reflects the degree of fit between predicted and actual traffic flow. Given that MAPE values are generally higher for models in mid-altitude and low-altitude airspaces, we focus on these two airspaces to visualise the actual traffic flow alongside predictions from state-of-the-art models.

**Fig 8:**
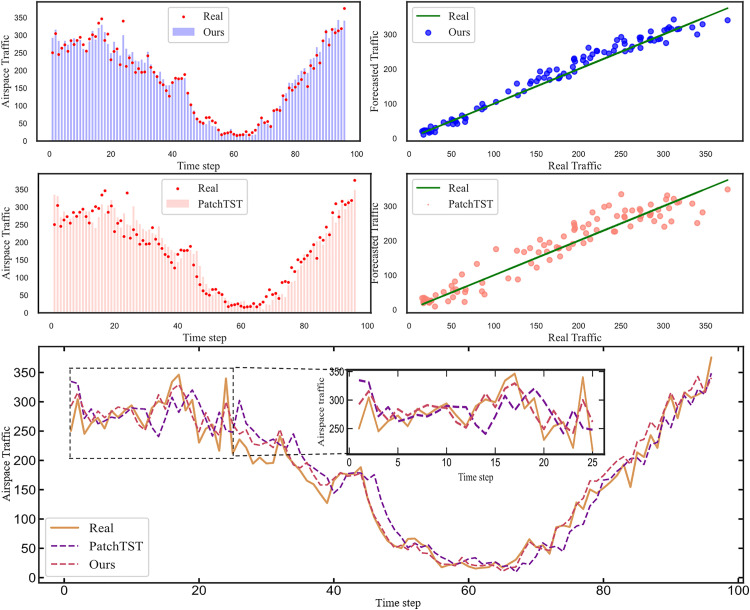
Plot of actual vs predicted flight traffic in Low-altitude airspace.

**Fig 9:**
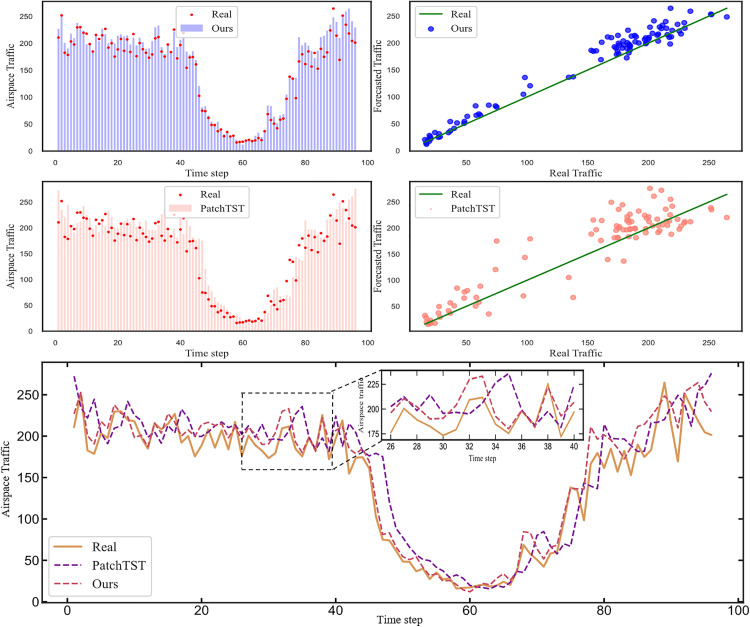
Plot of actual vs predicted flight traffic in Mid-altitude airspace.

Figs 8 and 9 offer a detailed visual comparison of the prediction performance of our proposed model and the PatchTST baseline. The top two rows of each fig illustrate the individual performance of our proposed model and PatchTST, respectively. For each model, two subplots are provided: a time-series plot on the left compares the predicted traffic (bar chart) against the actual values (red dots) to visualize the temporal fit, while a correlation plot on the right maps predicted values against actual values, where the proximity of the scatter points to the 45-degree line of perfect prediction indicates accuracy. The bottom panel then provides a direct time-series overlay, visualizing the predictions of both models as line graphs against the real traffic data, with an inset offering a magnified view of a specific interval to highlight performance on more volatile segments.

Figs 8 and 9 illustrate that flight traffic in the Mid-altitude and Low-altitude airspace exhibits significant fluctuations. These variations are primarily attributable to the impact of low-altitude fog, which reduces visibility and directly increases the intervals between take-offs and landings, thereby causing fluctuations in flight traffic data. In the Mid-altitude airspace, the influence of clouds differs from that at lower altitudes but remains substantial. Mid-altitude clouds are closely associated with weather systems, often heralding precipitation or storm systems. Such weather events prompt aircraft to alter flight paths or adjust altitudes, thereby introducing variability in mid-altitude airspace traffic.

Our model consistently demonstrates superior fit to actual traffic flow compared to the second-best model across all time intervals, with a notable advantage during periods of heightened prediction difficulty. This underscores the effectiveness of employing one-dimensional convolution to separately model trends and seasonality in air traffic flow. Additionally, the use of linear layers proves sufficient for capturing linear patterns within the data. These findings further validate the robustness of the proposed model in handling the complexities of dynamic airspace environments.

### Ablation studies

To evaluate the contribution of each component in the airspace traffic prediction task, a series of ablation experiments were conducted on five IDCformer variants using the traffic data of the entire airspace. These experiments systematically dissected the impact of key modules within the model. The variants are defined as follows:

IDCformer-RLAT: LAT module removed.
IDCformer-RL: Linear layer removed.
IDCformer-RC: A set of Conv1d in TSE removed.
IDCformer-RTSE: TSE module removed
IDCformer-RP: Enhanced PatchTST removed.

**Fig 10:**
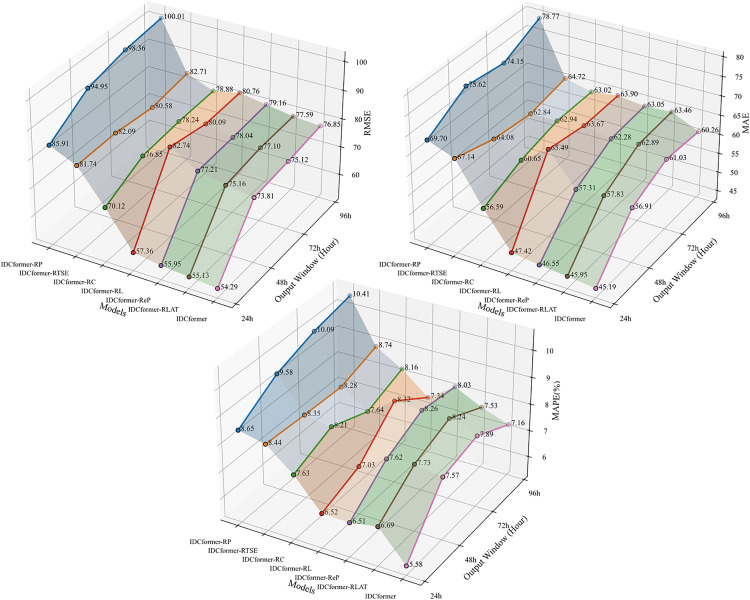
Experimental results after removing different modules.

**Fig 11:**
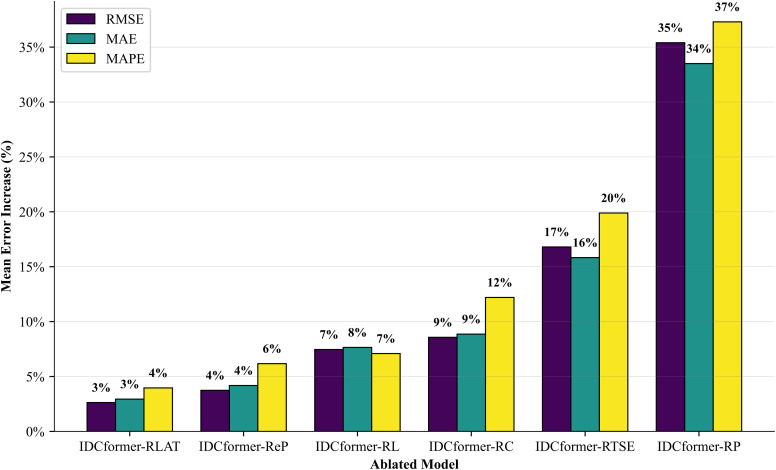
The mean error increase of ablated models compared to IDCformer.

The input variables for each model are listed in Table 3. The prediction evaluations are illustrated in Fig 10, demonstrating that each component contributes to the model’s predictive performance. Fig 11 depicts the mean error increase for each ablated model compared to the original model, showing that the removal of any component results in decreased model performance. Based on this, the following conclusions can be drawn:

**Table 3:** Input and output variables of the IDCformer and each variant.

Statistical indicator	Model	Input	Output
Airspace traffic	IDCformer	y(t−1),...,y(t−96),TSE,Linear,PatchTST,LAT	Y(t)
	IDCformer-RL	y(t−1),...,y(t−96),TSE,PatchTST,LAT	Y(t)
	IDCformer-RLAT	y(t−1),...,y(t−96),TSE,Linear,PatchTST	Y(t)
	IDCformer-RC	y(t−1),.,y(−96),Conv1d,Linear,PatchTST,LAT	Y(t)
	IDCformer-RTSE	y(t−1),...,y(t−96),Linear,PatchTST,LAT	Y(t)
	IDCformer-RP	y(t−1),...,y(t−96),TSE,Linear,LAT	Y(t)

1. Effectiveness of the Patching Technique and the Enhanced Position-Aware PatchTST: The results highlight the effectiveness of both the general patching technique and our specific enhancements. A comparison between IDCformer-RP (which removes the PatchTST module entirely) and the full model reveals that IDCformer-RP exhibits the poorest performance. This demonstrates that the patching technique itself is fundamentally important, as it efficiently encodes the structural features of the input data and provides essential contextual information for the Transformer encoder.Furthermore, to specifically evaluate the contribution of our proposed enhancements, we tested a variant where the Enhanced PatchTST was replaced by the original PatchTST. This variant also underperformed the full IDCformer model, confirming the value of our modifications. By applying patching to high-level feature representations and integrating implicit convolutional positional signals, our enhanced module provides the model with a more robust understanding of temporal context. This is particularly crucial in long-term forecasting as it helps prevent the loss of positional information over long sequences, leading to a significant improvement in prediction accuracy.
2. Effectiveness of TSE and LAT Modules: Effectiveness of TSE and LAT: As shown in Fig 10, a comparison of the errors among IDCformer-RTSE, IDCformer-RC, IDCformer-RLAT, and the full IDCformer model demonstrates that the model’s error increases significantly, whether TSE is incrementally removed or LAT is directly removed.This phenomenon arises because the convolutional operations within TSE facilitate hierarchical feature abstraction, aiding the model in recognizing higher-level patterns and traffic flow trends. Moreover, when the TSE module is removed, the positional information inherently encoded in the patches due to convolutional characteristics is also lost. This leads to ambiguity in sequence positional information, particularly for long-term predictions. The LAT module, on the other hand, effectively captures local dependencies and mitigates the influence of long-range noise. Consequently, the removal of either TSE or LAT substantially impairs the predictive performance of the model.
3. Effectiveness of the Overall Architecture: The full IDCformer model outperforms all variants, underscoring the pivotal role of its constituent modules. The high accuracy of IDCformer in long-term prediction is attributable not only to the superior performance of individual components but also to the synergistic integration of these components within the model architecture.

### Impact of different input_window

**Fig 12:**
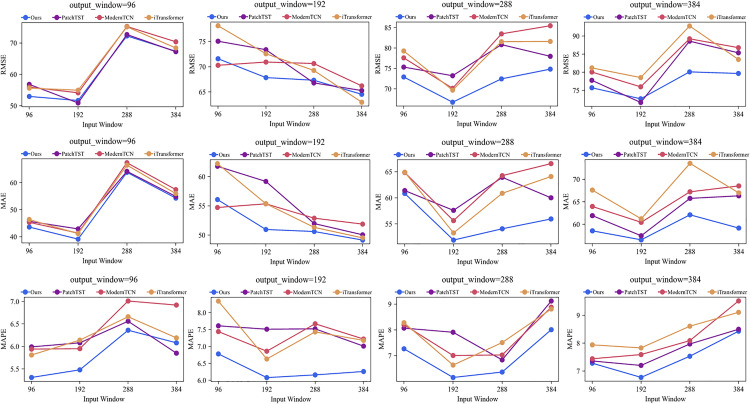
Performance of three error metrics (Y-axis) for different input_window (X-axis) on different output_window for Entire airspace traffic.

For deep neural networks, the input window (i.e., look-back window) is a key parameter that significantly affects the model’s ability to learn essential information from historical data. Selecting an appropriate input window can significantly improve the model’s prediction performance. Fig 12 demonstrates the error performance of various models on the entire airspace under different input window sizes. This analysis aims to evaluate how different input window sizes affect the model’s prediction performance while maintaining a consistent output window (i.e., prediction horizon).

Analysis by Different Metrics: Each row in Fig 12 represents a distinct error metric, specifically RMSE, MAE, and MAPE. It is evident from the figure that, irrespective of the metric utilized, the error of our proposed model consistently remains lower than that of the other state-of-the-art models as both input and output windows increase.

Analysis by Different Output Windows: Each column in Fig 12 corresponds to a different output window size. The performance of each model improves with increasing input window size only when the output window is set to 192. For output window sizes of 96, 288, and 344, the performance of each model peaks at an input window size of 192, after which it deteriorates as the input window size increases further. Consequently, an input window size of 192 is deemed optimal compared to other input window sizes. Additionally, we observed that the error of each model decreases when the input window is increased from 96 to 192. However, the prediction error increases sharply when the input window is expanded from 192 to 288 and 384. When the input window was set to 96, the models lacked sufficient information to accurately predict the changing patterns of flight traffic in future airspace. Conversely, an input window size of 384 introduced excessive redundant information into the model, leading to inaccuracies in predicting future traffic flows and thus diminishing model performance.

### Efficiency analysis

To evaluate the suitability of the proposed model for the airspace traffic forecasting task, we use the entire altitude dataset to analyze the computational cost of the proposed model. The experiments select training time, inference time, and GPU memory usage as the evaluation metrics, conducted in a unified environment with a fixed batch size of 64 to ensure comparability.

The results in Table 4 indicate that the MLP-based AMD is the most computationally efficient, followed by the iTransformer. Our proposed IDCformer, owing to its more complex, multi-stage architecture that includes dedicated modules for feature extraction (TSE) and local refinement (LAT), exhibits moderately higher computational costs. However, these costs remain well within a practical range for real-world deployment. For critical applications such as airspace traffic management, predictive accuracy is paramount to ensure operational safety and efficiency. As demonstrated in the preceding sections, IDCformer consistently achieves the highest forecasting accuracy. Therefore, the modest increase in computational resources is considered a justifiable and acceptable trade-off for the significant and reliable gains in predictive performance.

**Table 4:** Computational costs of different models on entire altitude dataset.

Model/Module	Training Speed(s/epoch)	Inference time(s/batch)	Memory Usage (MB)
IDCformer	0.8582	0.0082	108.3231
AMD	0.4137	0.0029	52.7856
iTransformer	0.6561	0.0045	95.4182

### The external information integration ability of IDCformer

Although the IDCformer proposed in this study is primarily designed to address the internal dynamics of airspace traffic data, the dynamic variations in flight traffic during actual air traffic operations are often influenced by multiple external factors. Therefore, this section focuses on evaluating whether IDCformer possesses the capability to effectively learn and integrate external information. Let the original feature vector at time t, containing internal traffic variables (e.g., number of flights), be denoted as $x_{t}^{internal}\in R^{d_{\text{internal}}}$. The selected external features (e.g., average flight altitude, longitude, and latitude) at the same time step are represented by another vector, $x_{t}^{external}\in R^{d_{\text{external}}}$. These two vectors are then concatenated to form a new, augmented feature vector, $x_{t}^{aug}$:


$$x_{t}^{aug}=x_{t}^{internal}⊕x_{t}^{external}\in R^{d_{internal}+d_{external}}$$ (17)

Where ⊕ denotes the concatenation operation. The model then uses the historical sequence of these augmented feature vectors, $x_{t}^{aug}$, as its new input to predict the future traffic count. This formulation allows the model to learn from both the intrinsic traffic dynamics and the external factors provided. The subsequent experiments analyze the impact of including these external variables.

**Fig 13:**
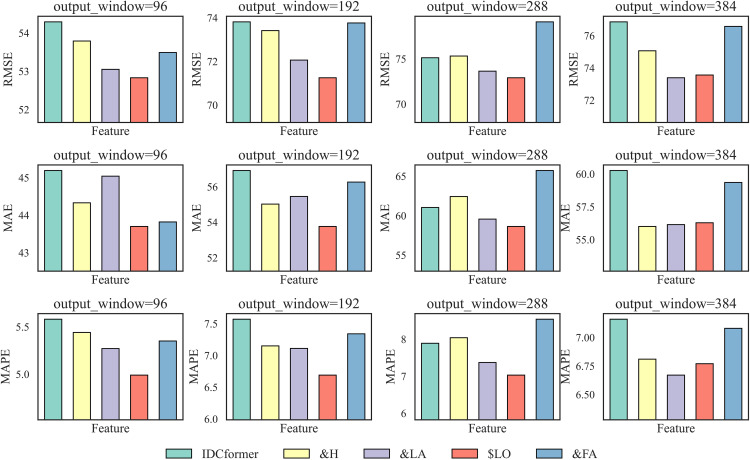
Effect of adding different features on the error of predicting Entire airspace traffic.

In this study, four external factors—aircraft heading, longitude, latitude, and flight altitude—were selected as features to analyze airspace traffic data. The prediction error of IDCformer was calculated, and Fig 13 illustrates the impact of these factors on long-term traffic prediction performance.

Characteristic Description and Rationale for Selection: Aircraft Heading represents the azimuth of flight relative to true north. The heading reflects the flight path of an aircraft, aiding in capturing the distribution characteristics of aircraft on different routes. Longitude and Latitude indicate the geographic coordinates in the east-west and north-south directions, respectively. These coordinates assist the model in capturing spatial traffic change patterns. Flight Altitude indicates the vertical distance of an aircraft relative to sea level. Changes in flight altitude directly affect route selection and airspace utilization. Incorporating these features is essential for a comprehensive understanding of the spatial and temporal dynamics of air traffic.

Impact of Adding Features: Analyzing Fig 13, it was observed that when the prediction duration is 72 hours (i.e., output window = 288), adding two external factors—flight altitude and heading angle—results in higher prediction errors compared to the original IDCformer model. Apart from this exception, the inclusion of any individual feature improved the model’s long-term prediction accuracy. This outcome highlights the model’s inherent capability to adaptively learn from external features. This adaptive learning is a direct result of the model’s hierarchical attention architecture. The global self-attention mechanism in the main Transformer encoder first assesses the overall importance of each external feature across the entire prediction horizon. Subsequently, the Local Self-Attention (LAT) module refines this understanding by focusing on smaller time windows, allowing the model to capture how an external factor’s influence might vary over shorter periods. This synergy between global and local attention enables the model to dynamically amplify the weights of consistently relevant features while suppressing those that introduce noise, thereby effectively learning from a complex, multi-feature environment.

Table 5 presents the prediction performance of the model after introducing different features, including the prediction error after combining latitude and longitude. This allows for a clear quantification of the performance improvements resulting from capturing various types of information. The abbreviations in Table 5 correspond to the following feature combinations: ‘Only F’ represents the baseline model using only traffic flow; ‘&H’ denotes the addition of aircraft heading; ‘&LA’ adds latitude; ‘&LO’ adds longitude; ‘&FA’ adds flight altitude; and ‘& LA & LO’ represents the combined addition of latitude and longitude. When adding heading as a feature, the prediction accuracy improved by only 2.05% on average compared to the baseline model. This limited improvement is attributed to the extensive coverage of the study area, the large number of flights, and the significant variation in heading angles, which may render heading less effective as a feature input.

**Table 5:** Performance of the model in predicting flight traffic over the entire airspace after adding different features. Where dark colored numbers are optimal and underlined numbers are sub-optimal.

output_window	24h			48h			72h			96h		
Metrics	RMSE	MAE	MAPE	RMSE	MAE	MAPE	RMSE	MAE	MAPE	RMSE	MAE	MAPE
Only F	54.29	45.19	5.58	73.81	56.91	7.57	75.12	61.03	7.89	76.85	60.26	7.16
&H	53.79	44.33	5.44	73.41	55.02	7.15	75.31	62.42	8.04	75.06	56.00	6.81
&LA	53.05	45.04	5.27	72.06	55.45	7.11	73.64	59.56	7.37	**73.39**	**56.14**	**6.67**
$LO	**52.83**	**43.70**	**4.99**	**71.25**	**53.76**	**6.69**	**72.91**	**58.63**	**7.03**	73.56	56.28	6.77
&FA	53.49	43.82	5.35	73.76	56.26	7.34	75.88	62.71	8.26	76.57	59.35	7.08
& LA & LO	52.96	44.09	5.03	72.52	55.07	6.74	74.65	59.85	7.41	73.88	56.81	6.92

In contrast, adding latitude and longitude features individually enhanced the model’s prediction accuracy by 4.04% and 5.91%, respectively. This suggests that changes in latitude and longitude significantly impact overall traffic flow, and our model effectively captures the relationship between spatial location and traffic flow changes. However, although flight altitude is a crucial parameter in aviation operations, its performance enhancement was relatively limited. This may be due to the high number of flights introducing randomness or insufficient correlation with traffic patterns, making it challenging for the model to extract effective information from the altitude feature to improve prediction accuracy.

When both geographic features, latitude and longitude, were input into the model, the improvement in predictive accuracy was not as pronounced as when they were added separately. This suggests that the design of flight routes leads to high correlation between longitude and latitude, resulting in multicollinearity between these features. Feature redundancy can impede the model’s ability to extract effective information and may introduce noise, thereby affecting prediction performance. Therefore, the correlation and information gain between features must be carefully considered during the feature selection process.

**Fig 14:**
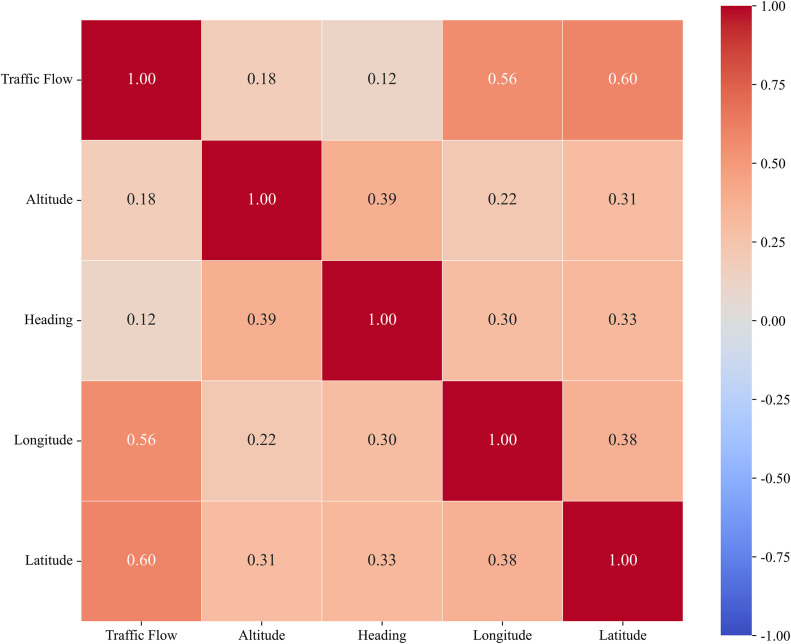
Pearson correlation heatmap of external features.

To succinctly quantify the contribution of each external feature and investigate the reasons for feature conflicts, we conducted a Pearson correlation analysis. Fig 14 presents the correlation matrix between the target variable (Traffic Flow) and external factors. The analysis yields two critical insights that explain the experimental results in Table 5:

Weak Correlation of Heading: The correlation coefficient between Heading and Traffic Flow is relatively low (r = 0.12). This confirms that while aircraft heading contains some valid information (contributing to the minor 2% performance gain), it is a weak predictor compared to spatial features, limiting its potential for significant improvements.

Feature Entanglement and Interference: A moderate correlation (r = 0.44) is observed between Longitude and Latitude. More importantly, we observe consistent cross-correlations between auxiliary features (Heading/Altitude) and spatial features (ranging from 0.30 to 0.39). This indicates a complex entanglement among external factors. When all features are integrated simultaneously, this coupling introduces inter-feature interference, which hinders the model from isolating unique information gains from each variable. This explains why the “combined” performance plateaued compared to individual feature integration.

In summary, our experimental results demonstrate that although IDCformer is specifically designed to capture the dynamic variations inherent in traffic data, it exhibits a robust capability to effectively learn and integrate external information. When external factors are incorporated as feature inputs, IDCformer achieves notable improvements in long-term prediction performance. Furthermore, the experiments reveal that individual independent factors are more conducive to enhancing the accuracy of long-term air traffic flow forecasting.

## Conclusion

This paper addresses the challenge of long-term airspace traffic forecasting by proposing a novel intrinsic dynamics capture network designed to overcome the limitations of traditional statistical models and machine learning methods in capturing complex dynamics and performing long-term predictions.

To enable in-depth analysis and modeling of airspace traffic data, we devised three core components: Trend and Seasonal feature Extraction (TSE), enhanced feature representation and position-aware PatchTST, and a Local Attention Mechanism (LAT). Experimental results on real-world datasets demonstrate the model’s mechanistic superiority. Specifically, the synergistic integration of TSE and LAT robustly addresses long-term dependency modeling by isolating and capturing multi-scale temporal dynamics. Simultaneously, the position-aware PatchTST effectively mitigates the loss of temporal order in extended horizons, ensuring precise positional information preservation. In terms of practical value for airspace management, IDCformer serves as a critical tool for strategic Air Traffic Flow Managemen. Its capability to accurately forecast long-term traffic peaks enables authorities to transition from reactive flow control to proactive congestion mitigation, facilitating data-driven decisions such as dynamic sector configuration and optimized controller staffing schedules, thereby enhancing both operational safety and efficiency.

Additionally, as internal variations in airspace traffic are influenced by external factors, we further investigated the model’s ability to integrate and learn from external information. Results show that incorporating independent external variables, particularly spatial positional information such as latitude and longitude, significantly improves long-term forecasting accuracy, validating the model’s applicability in real-world scenarios. This finding underscores the model’s dual capability to extract intrinsic data features while effectively learning spatiotemporal patterns. However, when multiple external factors are introduced simultaneously, the performance gains diminish compared to the inclusion of a single factor. This can be attributed to interference among external factors or the model’s suboptimal ability to capture the coupling relationships between different variables. This finding highlights the need for further optimization in model design to efficiently integrate the intrinsic patterns of traffic data with external information, aiming to achieve synergistic enhancements in predictive performance.

Future research will focus on enhancing the model’s predictive capability in response to sudden climate changes or abnormal conditions. We may also consider integrating additional dimensions of external factors and exploring optimal strategies for combining these factors to further improve the robustness and accuracy of airspace traffic prediction.
